# Morning versus Evening Intake of Creatine in Elite Female Handball Players

**DOI:** 10.3390/ijerph19010393

**Published:** 2021-12-30

**Authors:** Jose Manuel Jurado-Castro, Julián Campos-Pérez, M Ángeles Vilches-Redondo, Fernando Mata, Ainoa Navarrete-Pérez, Antonio Ranchal-Sanchez

**Affiliations:** 1 Metabolism and Investigation Unit, Maimonides Biomedical Research Institute of Cordoba (IMIBIC), Reina Sofia University Hospital, University of Cordoba, 14004 Cordoba, Spain; 2 Escuela Universitaria de Osuna (Centro Adscrito a la Universidad de Sevilla), 41640 Osuna, Spain; 3 Department of Food Science and Technology, Rabanales University Campus, University of Cordoba, 14071 Cordoba, Spain; m02capej@uco.es; 4 Department of Nursing, Pharmacology and Physiotherapy, Faculty of Medicine and Nursing, University of Cordoba, 14071 Cordoba, Spain; mariangelesvilches1@hotmail.com; 5 Centro de Estudios Avanzados en Nutrición (CEAN), 14010 Cordoba, Spain; fmataor@gmail.com; 6 Maimonides Biomedical Research Institute of Cordoba (IMIBIC), University of Cordoba, 14004 Cordoba, Spain; 7 Neuroplasticity and Oxidative Stress, Maimonides Biomedical Research Institute of Córdoba (IMIBIC), University of Córdoba, 14004 Cordoba, Spain; ep2napea@uco.es

**Keywords:** woman, female, sports training, sports performance, creatine, circadian rhythms, sports performance

## Abstract

A great deal of evidence has been gathered on the use of creatine as an ergogenic supplement. Recent studies show greater benefits when creatine ingestion is performed close in time to training, but few studies tackle the way that circadian rhythms could influence creatine consumption. The aim of this study was therefore to observe the influence circadian rhythms exert on sports performance after creatine supplementation. Our method involved randomly assigning fourteen women players of a handball team into two groups in a single-blind study: one that consumed the supplement in the morning and one that consumed it in the evening, with both groups following a specific training program. After twelve weeks, the participants exhibited a decreased fat percentage, increased body weight and body water, and improved performance, with these results being very similar in the two groups. It is therefore concluded that, although circadian rhythms may influence performance, these appear not to affect creatine supplementation, as creatine is stored intramuscularly and is available for those moments of high energy demand, regardless of the time of day.

## 1. Introduction

Dietary supplements are a common strategy for achieving improved health status and benefiting athletic performance [1]. Extensive research has been conducted on the different types of ergogenic dietary supplements used in sport and their benefit for performance, with creatine (Cr), particularly Cr monohydrate, being one of the most widely studied and one with the most evidence [2].

Cr is a compound that is synthesised in the liver, kidneys, and pancreas from the amino acids glycine, arginine, and methionine [3], but it can also be obtained through the diet by eating meat and fish, and it is also, in small amounts, found in some vegetables. Its absorption is favoured by the consumption of simple carbohydrates, and it accumulates mainly in skeletal muscle (95%), where 40% is in free form and 60% is in the form of phosphocreatine [4]. Under resting conditions, adenosine triphosphate (ATP) is formed in the mitochondria from adenosine diphosphate (ADP) through the process of oxidative phosphorylation. In muscles, ATP is used by the enzyme phosphorylcreatine kinase (CK) to convert Cr to Cr phosphate. This enzyme can reverse the reaction to obtain additional ATP, making Cr phosphate a temporary store of ATP under high energy demand conditions [5].

The importance of this compound lies in the fact that it provides energy when used in the resynthesis of ATP, giving it ergogenic potential (when consumed as a supplement), improving performance in athletes, specifically in high-intensity, short-duration exercise, increasing power and strength and improving body composition [4,5].

Although several studies have attempted to elucidate whether the best time to ingest Cr is before or after training [6], the results showed greater benefits when Cr was ingested close to the training sessions due to the increased blood flow, with significant improvements seen with post-training consumption [7] due to the fact that Cr can increase glycogen formation in the muscle and increase insulin sensitivity [8].

Another aspect to take into account when scheduling both training and sports supplementation is circadian rhythms. The time at which the physical activity is performed is another variable to consider, as a number of physiological changes occur that could affect sporting performance [9]. Therefore, based on the premise that an increase in body temperature seems to be strongly related to physical performance, the peak of body temperature coincides with the time of greatest activity and can cause variations in cardiorespiratory rate and muscle strength [10]. These variations are the result of the physiological, metabolic, and psychological rhythms synchronizing; the peak of these rhythms being in the early afternoon, when muscle hypertrophy increases due to increased hormone and growth factor binding protein levels (IGFBP-3). In addition to the increased muscle repair that results from elevated levels of creatine kinase and homocysteine, there are increased levels of antioxidant activity [9,11]. This may be influenced by the consumption of certain ergogenic substances used in sports supplements, which may enhance the ergogenic effect [12].

In view of the above, the aim of this study was to elucidate whether circadian rhythms could influence the ergogenic effect of Cr supplements by testing whether evening or morning intake improved athletic performance more in elite female athletes.

Therefore, it was hypothesized that Cr supplementation would improve the performance of female handball players regardless of a possible influence of the circadian rhythm.

## 2. Materials and Methods

### 2.1. Design

A randomized clinical trial was conducted on 14 female handball players competing in the highest national category. The study was designed according to the Consolidated Standards of Reporting Trials (CONSORT), with the appropriate adaptations (Table S1—CONSORT Checklist).

The effect Cr monohydrate supplements had on improving the performance of the players was evaluated by randomizing the sample into two groups, where half trained and took Cr in the morning (morning group) and the other half in the evening (evening group) for a period of twelve weeks, according to the protocol described below. All the variables were measured at baseline (week 0) and at the end of the intervention programme (week 12).

### 2.2. Participants

For this study, professional players were chosen from a women’s handball team playing in the top Spanish league in the 2020/2021 season.

The inclusion criteria were the following: (1) age between 18 and 35 years; (2) possession of a federation membership in their club; (3) not suffering from any type of illness or injury that would prevent them from participating in the study. These inclusion criteria were verified through personal interviews.

Taking into account the possible influence an altered hormonal secretion could have on circadian rhythm, and that menstrual cycle could have influenced the sports performance of the players [9], information about the menstrual status of participants was collected. They all presented physiologically normal periods without alterations. Furthermore, only one participant indicated taking oral contraceptives. The type of contraceptives was monophasic oral contraceptive with 3 mg of drospirenone and 0.02 mg ethinylestradiol.

#### 2.2.1. Ethical Aspects

The participants recruited were briefed on the protocol and objective of the study, and they signed a mandatory written consent prior to the start of the research. The study was conducted in accordance with the Declaration of Helsinki [13], and the project protocol was approved by the Cordoba Provincial Research Ethics Committee on 26 April 2021, with code ARS2921.

#### 2.2.2. Randomisation

The participants were randomly selected and assigned to two groups using the web page https://www.randomlists.com/team-generator (accessed on 30 April 2021): one group of which took the Cr supplement and trained in the morning and the other in the evening. The participation of a control group that did not consume the Cr supplement was not considered necessary given the high level of scientific evidence that exists on how Cr improves performance [2,14,15].

### 2.3. Intervention Procedure

The Cr monohydrate supplement was distributed to the participants by one of the researchers who explained how they should take it. They were instructed to dilute it in 250 mL of water and consume it after strength training according to their assigned group (morning or evening) or at the same time if they had a rest day.

The intake protocol consisted of a 5-day loading phase with a standard intake of 0.3 g·kg^−1^·day^−1^, followed by a maintenance phase with 0.03 g·kg^−1^·day^−1^ [8] after morning or evening training according to the assigned group, in order to achieve higher phosphocreatine reserves in skeletal muscle [15].

### 2.4. Training Protocol

In terms of training, all the participants generally carried out specific technical–tactical handball training sessions five days a week, each lasting an hour and a half. In addition, the participants underwent specific strength training and performed in the morning or in the evening, depending on the assigned group, supervised by a Physical Activity and Sports Science technician three times a week for at least one hour in which they worked on a “full-body” routine, performing 4 sets of 12 repetitions at 70% of one repetition maximum (1RM) of the following exercises: squats, bench press, dead weight, front pull-up, and military press. The 70% 1RM was estimated with a linear position transducer (encoder) (Speed4Lift v.4.1, Speed4Lift, Madrid, Spain) during the warm-up. In addition, at the weekend they played a competitive match lasting one hour (30 min each half). This frequency of training and matches was carried out throughout the season, regardless of the intervention.

### 2.5. Dietary Guidelines

As the diet of the players could affect energy metabolism during exercise, the participants were given nutritional guidelines to ensure that, during the study, they followed a dietary pattern with the following macronutrient distribution: 5.0 g·kg^−1^ fat-free mass·day^−1^ carbohydrate, 2.5 g·kg^−1^ fat-free mass·day^−1^ protein, and 1.0 g·kg^−1^ fat-free mass·day^−1^ fat [16]. Before starting the intervention, a nutritional session was carried out with the guidelines of the diet by exchanges. They were given tables of food groups and rations per exchange, calculated from their body weight. Thus, for 10 g of each macronutrient, there was 1 exchange of the food groups that contained this macronutrient in the majority, according to Russolillo et al. [17]. They were also explained the nutritional tool The Athlete’s Plate, a guide to sports meals created by dietitians of the United States Olympic Committee. With this tool, the athletes could modify the size of portions and servings of each food group according to the duration and intensity of their training [18]. All participants followed an omnivore dietary pattern and a Mediterranean diet.

Moreover, all the participants were instructed to refrain from consuming other ergogenic substances while they were participating in the study. During training sessions and matches this could be verified by the researchers.

### 2.6. Study Variables

#### 2.6.1. Body Composition and Anthropometric Measurements

The anthropometry and body composition of all the participants were measured at the beginning and end of the study, following the protocol established by the International Society for the Advancement of Kineanthropometry (ISAK) [19]. The participants’ height was measured (Seca 214 portable stadiometer; Seca, Hamburg, Germany), as was their body composition, using bioelectrical bioimpedance analysis (Tanita MC-780MA; Tanita Corporation, Tokyo, Japan). The participants received specific indications for the standardization of the measurement [20].

Arm circumference was measured using a non-elastic flexible tape measure (Cescorf Scientific model, sensitivity 0.1 mm, Rio Grande do Sul, Brazil) and skinfold thickness with a plicometer (Holtain DIM-98.610ND, sensitivity 0.2 mm, Crymych, UK), measuring in defined areas, always avoiding muscle. The anthropometric measurements consisted of three skinfolds and the circumference of the triceps muscle of the dominant arm, with this at rest and parallel to the body [20]. All the anthropometric measurement data were collected by an ISAK certified technician (J.M.J-C.) with a technical measurement error of 0.57%. The technical error of measurement was within 5% agreement for skinfolds and within 1% for circumferences.

#### 2.6.2. Lower Body

##### Back Squat Muscle Strength (One Repetition Max Test)

The 1RM test for the back squat was assessed on a Smith machine (Technogym, Barcelona, Spain), which ensured verticality. Each participant stopped after the eccentric phase (between 1 and 1.5 s), with the bar resting on a support that limited the countermovement, allowing greater control and reproducibility of the measurement in the concentric phase. The protocol used has been described previously [21].

The participants were instructed to refrain from any exercise other than their daily activities for at least 72 h before the measurement tests.

All the participants performed a general warm-up prior to the test, consisting of 7 to 10 min of light to moderate cardiovascular exercise until 75% of maximum heart rate was reached and maintained (Polar H10, Kempele, Finland). The players then performed an exercise-specific warm-up set for 12 to 15 repetitions at approximately 40% of the participants’ perceived 1RM, with a load progression for each exercise of 3 to 6 load increments. Increments at each load were approximately 10% of 1RM until an average propulsive speed of 0.5 m·s^−1^ was reached, followed by increments of 5 to 10 kg until 1RM was achieved. The speed was controlled by means of a linear position transducer (encoder) (Speed4Lift v.4.1, Speed4Lift, Madrid, Spain), with a coefficient of variation (2.61%) with respect to the gold standard (V120: Trio; OptiTrack, NaturalPoint, Inc., Corvallis, OR, USA) [22].

The participants were urged to perform at their maximum speed in the concentric phase of each repetition to ensure the use of maximum muscle strength. A rest interval of three to five minutes was allowed between each successive attempt. For the test to be considered successful, each participant stood with their feet shoulder width apart and the bar at their shoulder blades with their hands gripping the bar, then flexed their knees to 90°, followed by extension to the original standing position [23]. The technique was observed by the researchers to verify that the exercise was being carried out correctly.

##### Power (Countermovement Jump)

After a three-minute rest, a specific warm-up consisting of three countermovement jumps (CMJ) at a moderate intensity (60–70% of perceived maximum performance) was performed. Subsequently, with two minutes rest in between, three CMJs were performed, with a recovery period of 45 s between jumps, observed by an evaluator who stood at a distance of 1.5 m in the frontal plane to monitor correct execution of the jump and to record the maximum height (cm) reached in the three attempts [24]. The participants were instructed to start each jump in a squatting position, with their knees bent at a 90° angle, while keeping their hands on their hips with their trunk upright, taking care not to interrupt the movement from the start of the jump to the end. The height reached was recorded using an infrared measurement sensor (ADR jumping, Ciudad Real, Spain) provided by the Department of Nursing, Pharmacology, and Physiotherapy at the Faculty of Medicine and Nursing (University of Cordoba).

#### 2.6.3. Upper Body

##### Muscle Strength (Medicine Ball Throws)

To assess the strength of the extensor muscles in the upper limbs, a standing medicine ball throw test was used (weight of ball: 5 kg). Prior to the start of the test, a two-minute warm-up was performed involving joint mobility exercises (flexo-extension and shoulder circumduction) as well as three ball throws at submaximal intensities (40, 60, 80% intensity, respectively). For this technique, the participants had to stand behind a line with their feet shoulder-width apart and throw the ball with both hands behind their heads. To perform this throw correctly, they had to bend their legs and extend their trunk to give themselves momentum, as well as extending their heels without taking their feet off the ground. This test was repeated three times, with a 30 s break in between, with the distance achieved (cm) for each throw being noted [25].

##### Grip Strength (Dynamometry)

Using a calibrated handgrip dynamometer (Takei TKK 5001, Takei Scientific Instruments Co. Ltd., Niigata, Japan), three maximal voluntary isometric contractions were measured to determine grip strength with the right and left hands, respectively [26]. To measure this correctly, the participants had to stand with their arm parallel to the body with their hand in a neutral position.

### 2.7. Sample Size

As the 1RM back squat was one of the main outcomes for this study, the sample size was determined by calculating the statistical power based on a previous study [27], with a power of 0.80 and a two-tailed α level set to 0.05; the minimum number of participants required to detect a 10% difference in 1RM back squat performance was estimated as 14.

### 2.8. Statistical Analysis

We ran a Shapiro–Wilk test for normality of variables and Levene’s test for equality of variances with a normal distribution of two groups as result. We then compared the mean results between the baseline measurements for the two groups (morning and evening) using a Student’s t-test. To compare baseline and final measurements, a repeated-measures test with the group as a fixed factor was carried out. The effect size (ES) of the repeated measures test was calculated using partial eta squared ($\eta_{p}^{2}$), with small considered to be under 0.25, medium as 0.26–0.63, and large above 0.63 [28]. A difference-in-difference (DD) analysis was performed to compare the changes in the intervention between the morning and evening groups. For the results to have practical significance of DD analysis, the relative ES was calculated as Hedge’s g [29] with its corresponding confidence interval (CI). The ES was considered to be large (ES > 0.8), moderate (ES = 0.8 to 0.5), small (ES = 0.5 to 0.2), or trivial (ES < 0.2). The results are expressed as the mean ± the standard deviation or the mean relative differences (∆). The level of statistical significance was set as *p* < 0.05. All the statistical treatments were carried out using SPSS software version 25 (IBM Corp. IBM SPSS Statistics for Windows, Version 25.0 Armonk, New York, NY, USA).

## 3. Results

**Figure 1:**
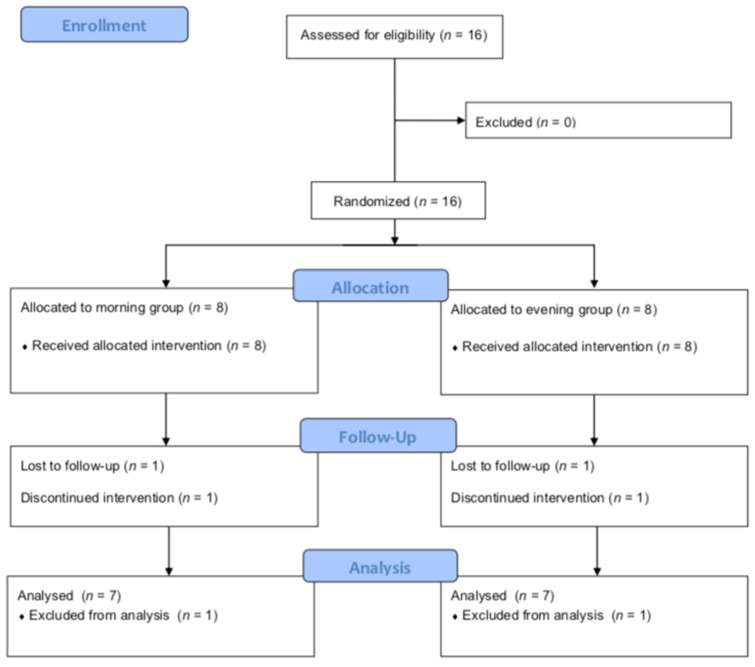
Flow Diagram CONSORT.

Fourteen female players completed the study and were included in the analyses, seven in the morning group (25.71 ± 3.90 years; 173.86 ± 6.47 cm) and seven in the evening group (22.71 ± 3.90 years; 169.43 ± 7.55 cm). Two participants (one in the morning group and one in the evening group) decided not to proceed with the intervention (Figure 1).

No significant differences (*p* > 0.05) were observed in body composition or in any study variable between the two groups (morning vs. evening) at the baseline (Table 1).

After the 12-week intervention period, a reduction in body fat percentage was observed in the morning group, as well as a reduction in the tricipital skinfold in the same group. Improved performance between the initial assessment and the end of the study was also observed in the two groups, with improvements in strength (1RM squat) and lower body power (CMJ), as well as medicine ball throwing. However, no improvement was observed in the upper body results associated with the dynamometer test (Table 1).

No differences were observed in the DD analysis between the morning and evening group for any of the variables studied (Table 2).

## 4. Discussion

The aim of this study was to investigate whether circadian rhythms influence Cr monohydrate supplementation in order to observe whether morning or evening intake of Cr monohydrate has a greater effect on the performance of elite female handball players with respect to the variables studied.

The results of this study indicate, in terms of the physical tests, that Cr monohydrate supplements improved performance in the 1RM squat, CMJ, and medicine ball tests in both groups (morning and evening), which was to be expected due to the already demonstrated effects of Cr on sports performance [1]. Although no differences in absolute values were found between the morning and evening groups, the evening group achieved a superior performance with respect to the morning group (1RM squat: 14.36 vs. 12.86; CMJ: 2.13 vs. 1.81; ball throwing: 0.28 vs. 0.25). Although performance increased in the above tests, no statistically significant differences were observed in the dynamometer test. These latter results are not in line with the published literature, given that various studies report an improvement in hand grip strength after Cr intake [30], which could be due to the type of sport, the time of the study, or the training program followed by our participants. Other studies, in turn, argue that grip strength is closely related to hand length [31], and given that this is not affected by Cr intake, this could explain why there is a discrepancy between our results and the existing literature. It would be worthwhile further investigating the importance of Cr intake in the improvement in handball dynamometry test results, particularly given the importance of the ball catching manoeuvre in this sport.

Studies such as those by Bonilla et al. [5] and Mills et al. [32] used similar variables to those in this study to determine what effect Cr supplements had on athletic performance. The results obtained in the present study are in line with those published in these two articles, with improvements being found in the 1RM squat, CMJ, and medicine ball throwing tests. With respect to the study by Chirosa-Ríos et al. [33], no significant differences were observed in the CMJ, which could be due to factors such as the type of training or the fact that there was no pre-loading phase. Given that our research involved female athletes, the results are within what would be expected according to the information found in various studies on performance improvement for both sexes [3,32,34] as well as in others carried out specifically on women [35,36,37], work showing increased muscle strength and higher load volumes in women after ingesting Cr.

In terms of body composition, the results of the study show a reduction in the percentage of body fat in both the morning (−4.88 ± 3.36) and evening groups (−2.38 ± 6.34), with a greater reduction in the percentage of adipose tissue in the morning group. This reduction in body fat percentage was accompanied by a reduced arm circumference (morning: −0.59 ± 0.92; evening: −0.83 ± 1.21), as well as the tricipital skinfold (morning: -7.75 ± 5.60; evening: −2.53 ± 3.97), in both groups, with the tricipital skinfold improvement once again being greater in the morning group. This may indicate that creatine supplements, in conjunction with a strength training programme, can improve body composition by reducing fat tissue without negatively affecting muscle mass. However, taking into account the difference in body composition results between the two groups, as well as the previous scientific evidence, which does not suggest that creatine alone can help weight loss, these reductions in fat percentage, tricipital fold, and arm circumference may be due to factors other than creatine, such as the training program followed, in addition to the possible influence of the time of training and of the dietary recommendations that were given to the players, which would explain the differences between the two groups.

Indeed, with respect to the influence of circadian rhythms, it is known that there is an individual biological preference for activity and rest influenced by chronotype (morning, intermediate, and evening), which is of particular importance when trying to improve performance in athletes [9]. Changes in performance related to factors that depend on circadian rhythms, such as temperature, blood pressure, energy metabolism, and hormone secretion, among other physiological variables, have been reported in the literature [10]. In relation to these changes, the increase in body and muscle temperature that occurs in the afternoon has been observed to improve ATP-PC and glycolytic ATP turnover, leading to greater muscle activation and strength [11].

Numerous studies argue that athletes perform better when training in the afternoon and that this is the result of the synchronization (chronotype) between physiological, psychological, and metabolic rhythms, which reach their peak in the early afternoon, in coordination with cardiovascular processes, which also show a circadian pattern [9]. This fact makes it reasonable to think that taking Cr supplements in the afternoon could be beneficial.

However, the results obtained show that no significant differences were found between the consumption of Cr supplements in the morning or in the evening. This lack of difference could be explained by the fact that Cr supplementation aids the intramuscular storage of Cr, keeping it available in case it is needed when there is a greater energy demand [38], whether this is in the morning or the evening. In addition, other studies have reported limited changes in performance with respect to the time of day the supplement is taken [39], and likewise, no differences have been demonstrated in terms of recovery from anaerobic exercise in the morning vs. the evening [11]. These results suggest the need for further studies on diurnal fluctuations and exercise performance, especially in well-trained athletes.

This study included some limitations, some of them being that there was no control group to compare the effect of Cr, so the results should be interpreted with caution. However, Cr has been successfully and extensively shown to be effective in enhancing athletic performance [2,15]. Moreover, this study did not have a daily dietary monitoring on the nutrition of the athletes, therefore being another limitation of the study. In future studies that compare the influence of the circadian rhythm and timing in supplementation and elite athletes, it would be interesting to carry out a nutritional monitoring that guarantees the dietary pattern. The consumption of protein, especially consumption of animal protein, is a covariate of special importance for future studies that delve into moments of Cr ingestion as a supplement for elite athletes.

Another limitation of our study was the relatively small size of the sample. Although clinical trials with a larger sample size are needed to confirm these findings, we can state that the results of the present study are in line with recent research on the optimal timing of Cr intake, which suggests that greater performance is achieved when Cr supplementation is performed after strength training [6,8,38]. Factors that could have influenced the different results obtained are the level of training of the participants and the initial amount of Cr present in their muscles. Given that the initial level of Cr in the muscles was not measured in this study, an attempt was made to correct any bias by carrying out a prior loading phase to try to homogenize the sample and ensure that all the participants started with similar levels of Cr reserves.

On the other hand, future research could focus on how the menstrual cycle affects Cr metabolism in female athletes and the repercussions this has on their performance [40], with a view to analysing whether Cr supplementation could achieve greater benefits and make up for the energy deficit that is allocated to other physiological processes in order to use it to improve performance.

## 5. Conclusions

In conclusion, the results confirm the ergogenic effect of Cr supplements in improving sporting performance in elite female handball players according to the specific physical tests of lower limb and upper limb strength carried out. Handball is a sport that has short duration and high intensity actions, such as direction changes, jumps, and throws, where the phosphocreatine system plays an important physiological role in the generation of ATP. The specific improvement in the strength and power of the lower limbs is important in many of these actions where this musculature is required for jumping or repeated high intensity sprints.

However, no significant differences in performance were observed between morning vs. evening intake of Cr following the supplementation protocol in the sample of players analysed.

## Figures and Tables

**Table 1:** Changes in body composition and sports performance variables in the morning and evening groups of female handball players after 12 weeks of intervention.

Variables	Group	Measurements		Δ	$\eta_{p}^{2}$
		Baseline (Week 0)	Final (Week 12)		
Weight (kg)	Morning	65.33 ± 5.99	65.66 ± 4.96	0.33 ± 1.69	0.045
	Evening	63.27 ± 9.10	63.40 ± 9.72	0.12 ± 1.45	0.009
BMI	Morning	21.91 ± 1.62	22.08 ± 1.12	0.16 ± 0.54	0.104
	Evening	22.02 ± 2.65	22.11 ± 2.56	0.09 ± 0.52	0.033
% Fat	Morning	27.43 ± 2.40	22.55 ± 4.21	−0.88 ± 3.36 *	0.717
	Evening	27.22 ± 5.79	24.50 ± 4.61	−2.38 ± 6.34	0.202
Lean Body Mass (kg)	Morning	47.38 ± 4.25	48.36 ± 4.35	0.97 ± 2.67	0.138
	Evening	46.12 ± 8.47	45.62 ± 6.25	−0.49 ± 2.41	0.048
% Body Water	Morning	51.46 ± 3.27	57.60 ± 3.24	6.13 ± 2.37 *	0.889
	Evening	50.31 ± 2.89	55.54 ± 3.36	5.22 ± 3.11 *	0.767
Arm Circumference (cm)	Morning	27.30 ± 1.61	26.70 ± 0.82	−0.59 ± 0.92	0.332
	Evening	27.52 ± 2.53	26.69 ± 2.98	−0.83 ± 1.21	0.353
Tricipital Skinfold (mm)	Morning	17.86 ± 5.81	10.10 ± 1.74	−7.75 ± 5.60 *	0.697
	Evening	14.17 ± 4.60	11.64 ± 3.07	−2.53 ± 3.97	0.321
1RM Saddle Squats (kg)	Morning	98.80 ± 22.86	112.79 ± 23.35	13.98 ± 12.86 *	0.579
	Evening	89.95 ± 12.32	104.01 ± 23.30	14.05 ± 14.36 *	0.527
CMJ (cm)	Morning	36.11 ± 6.07	38.32 ± 5.54	2.21 ± 1.81 *	0.635
	Evening	35.22 ± 6.86	37.81 ± 8.16	2.58 ± 2.13 *	0.631
Ball Throw (m)	Morning	4.98 ± 0.34	5.35 ± 0.50	0.37 ± 0.25 *	0.709
	Evening	4.66 ± 0.34	5.03 ± 0.44	0.37 ± 0.28 *	0.683
Dynamometer (kg)	Morning	36.57 ± 5.14	37.42 ± 5.28	0.85 ± 1.64	0.241
	Evening	33.66 ± 5.67	34.08 ± 5.06	0.41 ± 0.99	0.174

BMI, body mass index; 1RM, repetition maximum; CMJ, countermovement jump; $\eta_{p}^{2},\;\mathrm{partial}\;\mathrm{eta}\;\mathrm{squared};$ Δ, change between baseline and final measurement; * Indicated statistical significance between baseline and final measurement (*p* < 0.05).

**Table 2:** Comparison of body composition and sports performance variables in the morning vs. evening group after a 12-week intervention in female handball players.

Variables	Mean (Δ1–Δ2)	Difference in Differences (DD) *	ES
Weight (kg)	0.33 to 0.13	0.20	0.645
BMI	0.17 to 0.09	0.08	0.909
% Fat	−4.88 to −2.72	–2.16	0.387
Lean Body Mass (kg)	0.98 to −0.50	1.48	0.235
% Body Water	6.14 to 5.23	0.91	0.632
Arm Circumference (cm)	−0.60 to −0.83	0.23	0.461
Tricipital Skinfold (mm)	−7.76 to −2.53	–5.23	0.116
1RM Saddle Squats (kg)	13.99 to 14.06	–0.07	1.000
CMJ (cm)	2.21 to 2.59	–0.38	0.549
Ball Throw (m)	0.37 to 0.37	0.00	0.970
Dynamometer (kg)	0.85 to 0.42	0.43	0.617

BMI, body mass index; 1RM, one repetition maximum; CMJ, countermovement jump; Δ1, change between baseline and final measurement of the morning group; Δ2, change between baseline and final measurement of the evening group; DD, difference-in-differences. * No differences were observed in the DD analysis between the morning and evening group for any of the variables studied.

## Supplementary Materials

The following are available online at https://www.mdpi.com/article/10.3390/ijerph19010393/s1, Table S1: CONSORT 2010 checklist of information to include when reporting a randomised trial.
